# An Electrochemical Impedance Spectroscopy-Based Technique to Identify and Quantify Fermentable Sugars in Pineapple Waste Valorization for Bioethanol Production

**DOI:** 10.3390/s150922941

**Published:** 2015-09-11

**Authors:** Claudia Conesa, Eduardo García-Breijo, Edwin Loeff, Lucía Seguí, Pedro Fito, Nicolás Laguarda-Miró

**Affiliations:** 1 Instituto de Ingeniería de Alimentos para el Desarrollo (IIAD), Universitat Politècnica de València, Camí de Vera s/n, 46022 Valencia, Spain; E-Mails: lusegil@upvnet.upv.es (L.S.); pfito@tal.upv.es (P.F.); 2 Centro de Reconocimiento Molecular y Desarrollo Tecnológico (IDM), Unidad Mixta Universitat Politècnica de València, Universitat de València, Camí de Vera s/n, 46022 Valencia, Spain; E-Mails: egarciab@eln.upv.es (E.G.-B.); edloe@upvnet.upv.es (E.L.)

**Keywords:** bioethanol, saccharification, electrochemical impedance spectroscopy, fermentable sugars, pineapple waste

## Abstract

Electrochemical Impedance Spectroscopy (EIS) has been used to develop a methodology able to identify and quantify fermentable sugars present in the enzymatic hydrolysis phase of second-generation bioethanol production from pineapple waste. Thus, a low-cost non-destructive system consisting of a stainless double needle electrode associated to an electronic equipment that allows the implementation of EIS was developed. In order to validate the system, different concentrations of glucose, fructose and sucrose were added to the pineapple waste and analyzed both individually and in combination. Next, statistical data treatment enabled the design of specific Artificial Neural Networks-based mathematical models for each one of the studied sugars and their respective combinations. The obtained prediction models are robust and reliable and they are considered statistically valid (CCR% > 93.443%). These results allow us to introduce this EIS-based technique as an easy, fast, non-destructive, and *in-situ* alternative to the traditional laboratory methods for enzymatic hydrolysis monitoring.

## 1. Introduction

The search for sustainable and environmentally friendly energy sources alternative to fossil fuels is raising the investigation of agro-industrial wastes as potential inputs for second-generation bioethanol production. In this sense, pineapple is generating a growing interest as its world production is steadily increasing and has reached 24 million tons in 2014 [1]. Nowadays, around 33% of its production is being processed, mainly by canning and juice industry [2] and its industrial waste (crown, pulp and peel), representing about 50% (W/W) of the total processed fruit [3], cannot be neglected. In addition, its bio-chemical composition reinforces the interest in this waste as a potential source for bioethanol production because of its high content of cellulose and hemicellulose [4,5,6].

In order to produce bioethanol from lignocellulosic biomass, it is necessary to hydrolyze cellulose (polymer of d-glucose units linked by β-1,4-glycosidic bonds) and hemicellulose (polymer of pentoses, hexoses and uronic acids) into fermentable sugars [7]. This is the most complex phase in the bioethanol production process and can be performed by chemical or enzymatic hydrolysis. The enzyme-based saccharification is more efficient than the chemical hydrolysis, showing higher selectivity and lower energy costs. On the contrary, it is particularly complex due to the mechanism of the enzymatic hydrolysis and the relationship between the enzyme and the substrate structure [8].

Nowadays, there are several complex laboratory techniques for the identification and quantification of sugars generated during enzymatic hydrolysis processes, such as gas chromatography, high performance liquid chromatography, and enzymatic methods, even though the latter are generally applied for the quantification of a single type of sugar [9,10]. These techniques are very precise and considered as a reference but they are slow, expensive, destructive, and require skilled labor to be conducted.

Over the last few years, several electrochemical-based techniques have been raising and nowadays they are showing promising results for the identification of chemical compounds in an easy, rapid, non-destructive, and online way. In this regard, EIS is one of the most remarkable ones. This technique allows the analysis of the properties of the materials by a successive application of alternate electric signals at different frequencies (sinusoidal voltage or current) in the test sample, the subsequent registration of the current or voltage responses within an electrochemical cell and the calculation of the impedance value for each signal [11,12]. EIS has been successfully applied in several fields such as medicine [13,14,15], materials science and engineering [16,17,18], water [19] and environmental engineering [20]. EIS has also been widely applied in food engineering: study of salt levels in food products [21,22,23,24] quality control of fish [25,26,27] and meat products [28,29], and novel food processes [30,31].

In these electrochemical techniques, an appropriate statistical treatment of the obtained data becomes fundamental because of its large size. In this sense, Principal Components Analysis (PCA) and Partial Leasts Squares (PLS) are quite usual and efficient but nowadays Artificial Neural Networks (ANNs) have been raised as very promising and alternative methods to conduct sample classifications and pattern recognition [32]. These methods are called neural networks because of their similarity to the way the human brain processes information [33]. ANNs, as a biological brain, have a set of neurons linked together in a complex way and are able to treat information in a multifunctional process. In addition, ANNs are able to learn in their training process in order to improve themselves and find the optimal conditions to work showing high flexibility and adaptive capacity. In addition, ANNs are being used in a wide range of applications such as electronic noses [34,35] and tongues [36,37] and they are showing very interesting prediction models in several fields such as water [38], food [39] and the environment [40,41].

According to this, the aim of the present work is to study the suitability of EIS-based techniques to identify and quantify fermentable sugars present in the enzymatic saccharification process of pineapple wastes for bioethanol production by an optimized prediction system.

## 2. Experimental Section

### 2.1. Raw Material and Sample Preparation

Pineapple fruits selection (MD-2 cultivar, Extra Sweet or Golden Sweet) was based on external factors such as the absence of injuries, ripeness and weight. In order to prepare the samples, pineapples were first washed in a sodium hypochlorite solution (0.1%) for 5 min. Next, the crown was removed, and the pulp was separated from the rest of the fruit by using a pineapple cutter. Peel and core (waste) were cut into smaller pieces and grinded in a blender (Solac Inox Professional 1000 W Mixer). The resulting product was then frozen and kept at −22 °C until the experiments were conducted.

### 2.2. Electrochemical Impedance Spectroscopy Equipment

**Figure 1:**
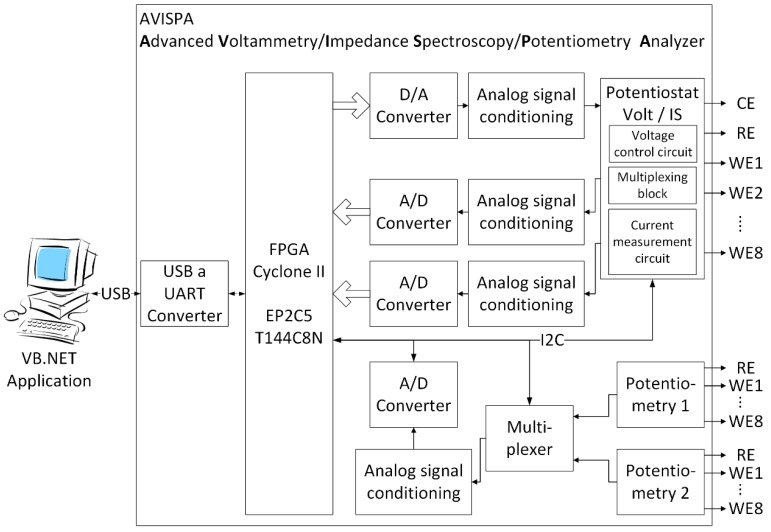
Block diagram of the AVISPA device.

EIS measurements were carried out using a system developed by the Group of Electronic Development and Printed Sensors (GED + PS) belonging to the Centro de Reconocimiento Molecular y Desarrollo Tecnológico (IDM) at the Universitat Politècnica de València (UPV). This system consists of a device called AVISPA (Advanced Voltammetry, Impedance Spectroscopy & Potentiometry Analyzer) (Figure 1) associated with a specific software application that is able to apply different sinusoidal voltage signals with amplitudes up to 1 Vpp and frequency sweep from 0.01 Hz to 10 MHz using up to 32 current scales. The hardware consists of an Altera Cyclone II EP2C5T144C8N Field Programmable Gate Array (FPGA), clocked at 100 MHz, a 12-bit THS5661A Digital-to-Analog Converter (DAC), two identical ADS6125 12-bit Analog-to-Digital Converters (ADC), and various analog blocks to adapt signals to the required levels. It also contains hardware to be able to select 32 current scales, by means of various shunt resistors, to increase the sensitivity of the current measurement.

The user can configure, by means of the software, the start and end frequency, the number of frequencies of the sweep and the amplitude of the sine wave to be generated. The user also has the option to fix the currents scale, or let the software choose the appropriate current scale at each measurement dynamically: if the values are below 20% or above 80% of the full range of the ADC, the software selects a higher or lower shunt resistor, respectively.

Once the measurement is started, the software calculates the digital values to be sent to the DAC, using a previously generated calibration file, and sends the data to a memory block inside the FPGA. Once the FPGA receives the last byte, it starts to generate the signal and acquire the data of the two ADCs simultaneously, which are written to two separate memory blocks. Once the signal generation and acquisition stage has been finished, the FPGA sends the obtained data to the PC software, where the digital data is converted to analog values by the use of a calibration file. The software calculates modulus and phase values and plots these values into a graph. This is repeated for each of the frequencies in the sequence obtaining a frequency response plot of the sample.

The sine wave generated is formed by 1000 points per cycle wherever allowed, taking into account the generated frequency and the working frequency of the FPGA. The worst case scenario is the generation of a 10 MHz signal that can only contain 10 points per cycle due to the clock speed of the FPGA at 100 MHz.

### 2.3. Electrochemical Impedance Spectroscopy Sensor

**Figure 2:**
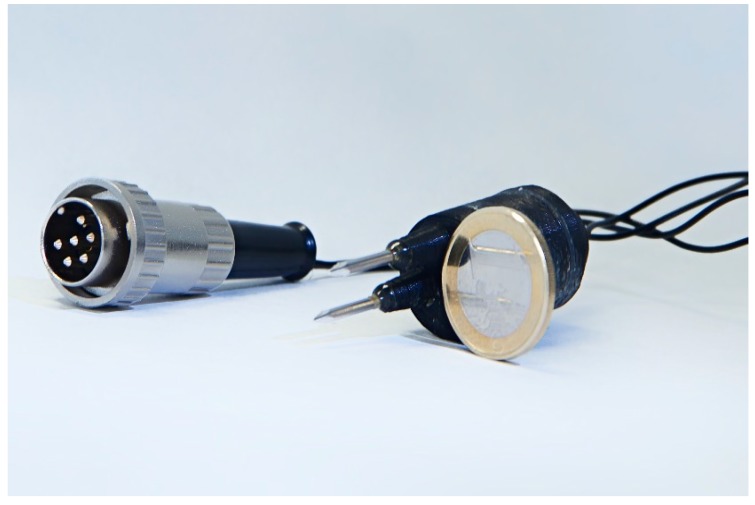
A view of the designed double needle electrode.

Previous studies suggested using non-oxidizable materials instead of oxidizable ones (e.g., Cu, Co, Ni, Ag) due to their rust resistance and easy handling, avoiding complex cleaning treatments of the electrodes [41,42]. Moreover stainless steel was selected among other non-oxidizable materials (e.g., Pt, Au, Ir, Rh) because of a clear economic motivation and its successful and extended use in food industry applications [21,28,30]. A double needle electrode (working and counter electrodes) composed of two parallel stainless steel needles 1.5 cm long and 1 mm in diameter, separated by a distance of 1 cm was used (Figure 2). The plastic frame containing the needles was designed with a 3D printer (EKOCYCLE™ Cube^®^, Cubify 3DSYSTEMS^®^) and fixed with an epoxy kit (RS 199-1468). This design keeps both a constant the separation between the needles and also a constant electrode surface in contact with the samples during the measurements as protects the electrical connections to transmit information to the device. The specific design of the sensor assures that the distance between electrodes is enough to consider a stable electric field preventing polarization effects. In addition, the use of parallel electrodes compared to other kind of designs (e.g., coaxial electrodes) generates a homogenous electric field distribution [22]; thus, an easier interpretation of the obtained measures is possible, so that the design is particularly appropriate for liquid samples.

### 2.4. Electrochemical Impedance Spectroscopy Measurements

EIS measures were conducted in thawed pineapple waste samples with pH adjusted to 5 by adding a few drops of NaOH 1N (Panreac Química, S.L.U.). The penetration depth of the electrodes into the samples was 1 cm and it was a constant for all the assays. Analyses were made in triplicate at 25 °C by using a thermostatic bath (PolyScience^®^) and samples were selected randomly in order to avoid any memory effect in the measurements.

First of all, individual identification and quantification of sugars was carried out taking into account that the absence of added sugars in some specific samples did not mean the absence of endogenous sugars in the raw material, which was considered as a baseline for these determinations. In addition, the presence or effect of any potential interfering compound in the samples was negligible due to the use of the same homogenous pineapple waste for all the analyses. Previous works in this research line determined the behavior of the existing sugars along the enzymatic hydrolysis in pineapple samples [43]. Thus, the concentration range for each sugar was selected attending to these results. Accordingly, seven different concentrations were added to the pineapple waste samples and then analyzed for each studied sugar: 0 g/L, 5 g/L, 10 g/L, 20 g/L, 30 g/L, 40 g/L, and 50 g/L for glucose, and 0 g/L, 5 g/L, 10 g/L, 15 g/L, 20 g/L, 25 g/L, and 30 g/L for sucrose and fructose. Analyses were conducted in triplicate for a total of 63 samples (189 analyses).

Next, identification and quantification of combined sugars was conducted. In order to assess the ability of EIS to identify and quantify combinations of three sugars, a total of 81 pineapple waste samples (241 analyses) were prepared by mixing the three studied sugars (glucose, fructose and sucrose) at three different added concentrations (0, 25 and 50 g/L).

Once the samples were thermostated and the AVISPA device was ready, EIS measurements started by placing the double needle electrode into the assayed sample. Then, the system carried out the procedure described in Section 2.2 in order to instantly show the modulus and phase of the signal on the PC screen and compile the data into the corresponding file for further analyses.

### 2.5. Statistical Analysis

PCAs were carried out with data obtained from the samples in order to assess the feasibility of the EIS technique to discriminate among different sugar concentrations both individually and in combination. PCAs were performed using just the specific impedance modulus and phase data obtained in the frequency range in which the sensor showed the highest sensitivity. In addition, PLS analyses were also carried out to create predictive models for each studied sugar from their respective EIS measurements. According to the literature and previous studies in this research line, PLS prediction models were created using two series of the experimental data (66% of the data for the calibration set). The model was then validated with the remaining series of experimental data (34% for the validation set) [41,44]. The accuracy was given by the root mean square error of prediction (RMSEP) and the coefficient of determination (*R*^2^). All multivariate analyses were performed using SOLO© (Eigenvector Research, Inc., Manson, WA, USA).

A commercial ANN software (Alyuda Neurointelligence 2.2^©^, Alyuda Research Inc., Los Altos, CA, USA) was used throughout this study in order to create alternative, flexible and more adaptive predictive models to PLS [35,38,39]. Multi-layer feed forward neural networks and a single hidden layer ANN structure were selected and on-line back propagation training algorithms were used for fitting the network.

The optimal network topology was selected by developing several artificial neural network structures in order to determine the number of neurons of the hidden layer. Similarly, several trials suggested the selection of logistic-type transfer functions for the output layer neurons and hyperbolic tangent-type functions for the hidden nodes. Random data division was used by Alyuda Neurointelligence 2.2^©^ in order to select the samples for training (70%), validation (15%) and test (15%) data [38,39,40]. In addition, overfitting was avoided by using proportional number of nodes in the network architecture [45], cross validation and early-stopping in the training phase, so that the difference between training and validation mean square errors was minimal. As described before, the accuracy of the model was given by the root mean square error of prediction (RMSEP) and the coefficient of determination (*R*^2^) in the case of numerical prediction models. On the other hand, when classification models were developed, the accuracy of the model was given by the correct classification rate (CCR%) and the confusion matrix.

## 3. Results and Discussion

### 3.1. Individual Identification and Quantification of Sugars

**Figure 3:**
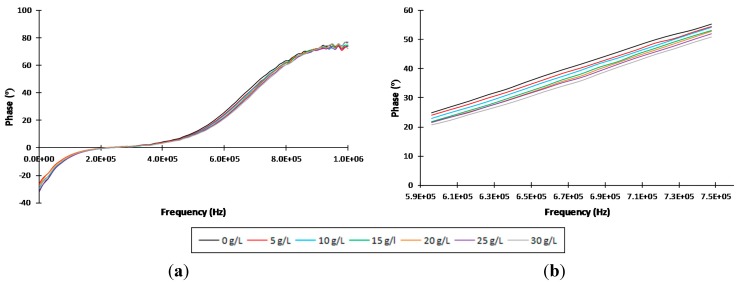
Averaged phase values of the impedance spectra of different sucrose concentration measurements for (**a**) the entire analyzed frequency range and (**b**) the selected range for data treatment (5.96 × 10^5^ Hz–7.47 × 10^5^ Hz).

The AVISPA device generated 200 data per analyzed sample corresponding to the modulus and phase of the 100 analyzed frequencies in each test. Analyses were carried out independently for each fermentable sugar at the above mentioned different concentrations. As shown in Figure 3, the frequency range showing the highest sensitivity to sucrose concentration was the one between 5.96 × 10^5^ Hz and 7.47 × 10^5^ Hz. Glucose and fructose showed their respective highest sensitivity in similar ranges. Consequently, this was the selected frequency range for data treatment and mathematical modeling.

**Figure 4:**
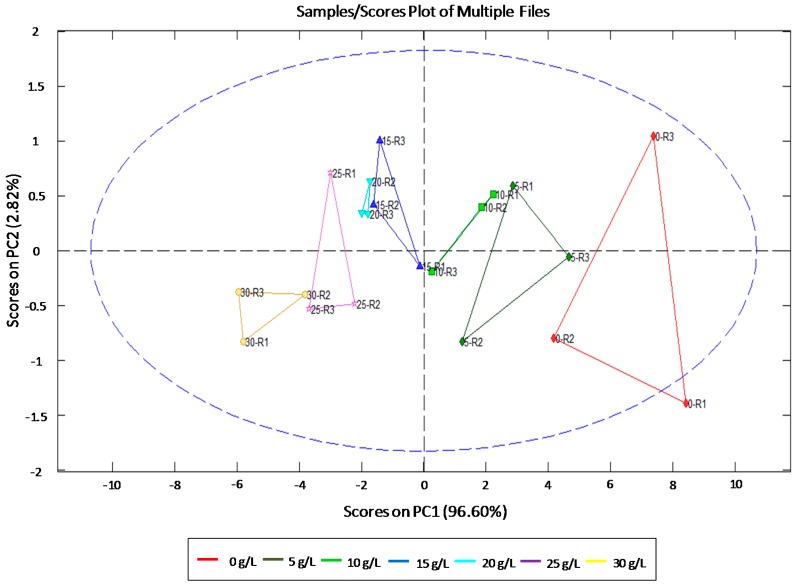
Principal component analysis (PCA) for the studied sucrose concentrations. R1–3: average of each replicate. The blue ellipsis indicates 95% confidence level.

PCA analyses showed a high percentage of the total variability (>99%) being explained just with the first two principal components for all the studied sugars. Specifically, variability for fructose data was explained up to 97.93% by the first component (PC1) and component 2 (PC2) explained the remaining 1.86% of the total variability. For sucrose, PC1 and PC2 explained 96.60% and 2.82% respectively of total variability (Figure 4). Finally, glucose variability was explained up to 97.57% and 2.19% by PC1 and PC2, respectively. Therefore, the results indicate that these concentrations can be discriminated with only one main component in the studied ranges.

Since PCA analysis showed that EIS analyses with the double needle sensor can discriminate different concentrations of glucose, fructose and sucrose, a PLS analysis was performed to predict these concentrations from EIS measures.

**Figure 5:**
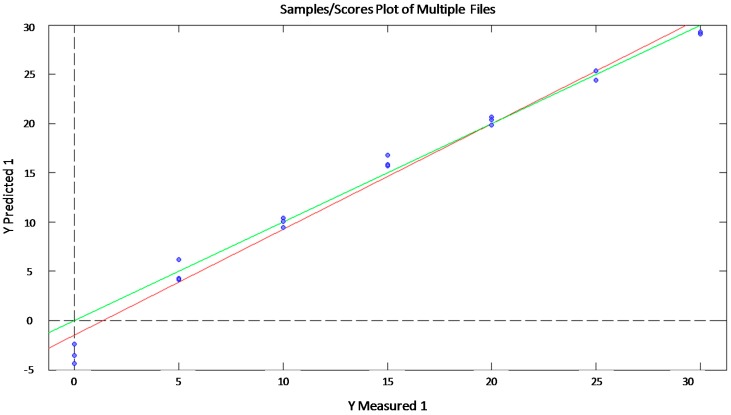
Correlation plot between experimental and predicted values of sucrose (g/L) by PLS statistical model (red line) and ideal behavior (green line).

As shown in Table 1, good correlations were obtained for all the analyzed sugars with *R*^2^ = 0.958 or above and RMSEP = 2.272 or below. These results demonstrated an accurate fitting between predicted and experimental values and, consequently, the obtained models can be considered statistically valid. The best correlation was obtained for sucrose as shown in Figure 5. Moreover, the PLS analysis for sucrose showed that a reliable mathematical model can be obtained using just one latent variable. Thus, the phase data for just one frequency is enough to quantify sucrose in a sample. Consequently, the prediction model could be very simple and accurate.

**Table 1:** Statistic values of Partial Least Square (PLS) discriminant analysis for the quantification of the studied fermentable sugars. (R^2^: coefficient of determination; RMSEP: Root Mean Square Error of Prediction; LV: Latent Variables.)

Sugars	Statistics		
	R^2^	RMSEP	LV
**Glucose**	0.979	2.272	3
**Fructose**	0.958	2.103	2
**Sucrose**	0.983	1.576	1

Consequently, the obtained results demonstrate that EIS is a robust and reliable methodology to quantify the concentration of the three main fermentable sugars in the studied ranges.

**Figure 6:**
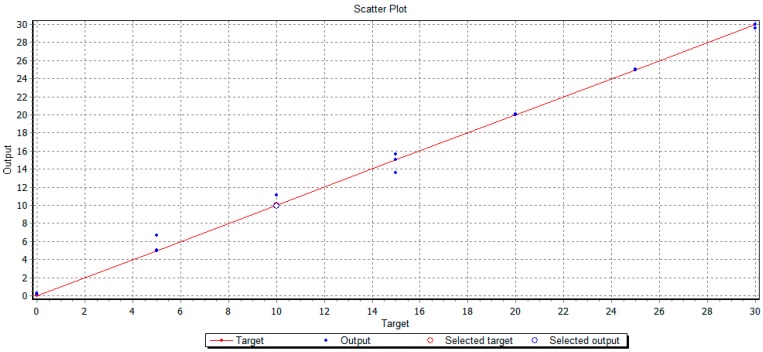
Regression line plot of the obtained ANN model for the studied sucrose concentrations (g/L).

However, as an alternative method to PLS analyses, Artificial Neural Networks-based models (ANN) were designed using the same data set. In order to do this, different net architectures were tested for each analyzed sugar to optimize the fitting between the EIS data and the expected response. Thus, a (16-8-1) architecture was designed for glucose that means 16 input nodes connected to an 8-node hidden layer and a final output layer. For fructose and sucrose, (16-21-1) and (16-2-1) architectures were selected. The training phase of these ANN generated mathematical models that are summarized in Table 2. The obtained models showed determination coefficients higher than 0.95 and RMSEP lower than 3.96. Figure 6 shows the regression line obtained by ANN for sucrose. These results demonstrate that the designed ANN model generates noticeable results with sufficient accuracy and reliability for modeling sugar concentration depending on the EIS response.

**Table 2:** Artificial neural network (ANN) results for the studied fermentable sugars (R^2^: coefficient of determination; RMSE: Root Mean Square Error).

		R^2^	RMSE
**Glucose**	**Training**	0.99	1.41
	**Validation**	0.88	3.39
	**Test**	0.95	3.96
**Fructose**	**Training**	0.96	1.39
	**Validation**	0.99	0.27
	**Test**	0.95	1.63
**Sucrose**	**Training**	0.99	0.09
	**Validation**	0.88	1.26
	**Test**	0.99	0.40

Considering R^2^ and RMSEP parameters for both PLS and ANN models, it follows that the fitting and accuracy of the models are quite similar (Table 1 and Table 2). However, slight differences in RMSEP are observed, so that a better fit for fructose and sucrose is obtained by ANN as glucose is better fit by PLS.

**Figure 7:**
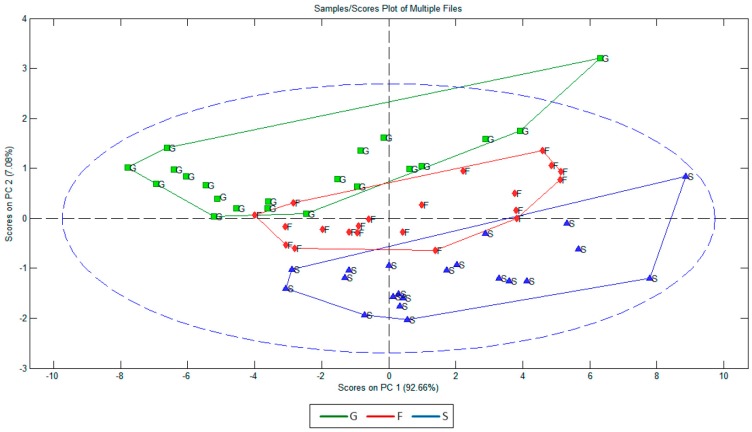
Principal component analysis (PCA) for the studied fermentable sugars: glucose (G), fructose (F) and sucrose (S).

Finally, a PCA analysis was performed to assess the ability of EIS to discriminate different fermentable sugars, comparing individual electrochemical responses in the studied frequency range (Figure 7). It is observed that the first two components explain a high percentage of the total variability (99.74%). Specifically, the first component (PC1) explains 92.66% and component 2 (PC2) explains the remaining 7.08% of the total variability. Therefore, the obtained results indicate that all three fermentable sugars can be easily identified with just one principal component using the electrochemical data in the studied ranges. It means that the phase data for just one frequency in the studied frequency range is enough to identify the kind of sugar in a sample. Consequently, the prediction model could be very simple and accurate.

### 3.2. Combined Identification and Quantification of Glucose, Sucrose and Fructose

Once the EIS technique and double needle electrode was demonstrated to be sensitive to the presence of fermentable sugars in pineapple waste samples, the next step was to assess its sensitivity to the combined presence of the three studied sugars. In order to do this, mixtures of these three sugars at three different concentrations (0 g/L, 25 g/L and 50 g/L) were analyzed in pineapple waste samples.

As in the previous cases, the AVISPA device generated 200 electrochemical data per assayed sample in the form of module and phase corresponding to 100 frequencies in the selected ranges. As happened before, phase data was the one showing the best sensitivity although in this specific case, the highest sensitivity was achieved in two different frequency ranges (1 Hz–1.41 × 10^5^ Hz) and (5.76 × 10^5^ Hz–8.48 × 10^5^ Hz).

Then, a PCA analysis was carried out in order to determine whether different combinations of the three fermentable sugars could be discriminated by EIS in the two studied frequency ranges. The result of this analysis showed that 97.29% of the total variability was explained with just two principal components as PC1 and PC2 explained 77.78% and 19.51% of the variability. Therefore, these results indicate that mixtures of the three studied sugars can be discriminated with just two principal components.

Next, PLS analyses were performed to generate a mathematical model able to predict concentrations of combined fermentable sugars. In order to do this, different PLS were conducted to check the capability of the system to detect and quantify each fermentable sugar from different mixtures of sugars in pineapple waste samples. The obtained results (*R*^2^ > 0.841 RMSEP < 8.23) indicate that PLS modeling for the combination of fermentable sugars is slightly lower than the ones shown in the previous cases. However, these results are not far from those obtained in other scientific studies in similar fields [39,41].

Therefore, ANN models were studied as an alternative to improve accuracy of the ones obtained by PLS. In this specific case, (11-38-9) was the selected ANN architecture to predict the combined concentration of the three fermentable sugars in pineapple waste samples.

The obtained ANN-based mathematical models generated very promising results showing CCR% values higher than 93.443% and confusion matrices like the ones shown in Table 3 for the combined quantification of the studied sugars. These results demonstrate that ANN-based models are remarkable complements to PLS models for predicting the combined concentrations of fermentable sugars in pineapple waste samples via EIS determinations, generating significant results with sufficient accuracy and reliability. Future studies will focus on studying the suitability of this EIS-based technique to monitor industrial saccharification and fermentation processes.

**Table 3:** Confusion matrices for combined sugars quantification.

**Glucose:** Mean CCR% = 93.443%			
Training	Validation	Test	Overall
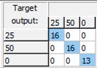	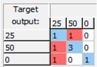	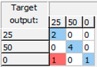	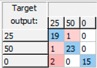
**Fructose:** Mean CCR% = 96.721%			
Training	Validation	Test	Overall
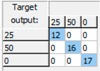	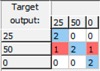	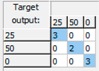	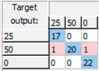
**Sucrose:** Mean CCR% = 100%			
Training	Validation	Test	Overall
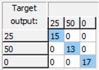	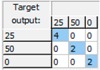	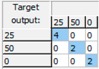	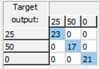

## 4. Conclusions

In the current energy outlook, the search for alternatives to fossil fuels is of strategic importance. In this sense, second-generation bioethanol production from agricultural and industrial food waste is a strategy that must be taken into account. Within this option, pineapple has a remarkable potential use due to its extensive worldwide market, the generation of an important waste volume in its industrial processing, and the bio-chemical composition of these wastes.

This work introduces an EIS-based methodology for monitoring and managing the concentration of sugars in the most complex phase for second generation bioethanol production: the enzymatic hydrolysis. In order to do this, an AVISPA device has been used as it is able to generate and receive EIS signals from an especially designed double needle sensor made of stainless steel. Statistical treatment of the data allowed to build reliable and robust ANN-based mathematical models (mean CCR% > 93.443%) to identify and quantify the main fermentable sugars (glucose, fructose and sucrose) in pineapple waste samples both individually and jointly. Furthermore, this methodology is easy, rapid, non-destructive, and *in-situ*. Thus, it can be considered as a promising alternative to the traditional laboratory techniques for enzymatic hydrolysis monitoring and management in second-generation bioethanol production not just from pineapple wastes but also from many other lignocellulosic sources.
